# Experiences and concerns of parents of children with a 16p11.2 deletion or duplication diagnosis: a reflexive thematic analysis

**DOI:** 10.1186/s40359-024-01609-9

**Published:** 2024-03-12

**Authors:** Charlotte E. Butter, Caitlin L. Goldie, Jessica H. Hall, Kathy Leadbitter, Emma M.M. Burkitt, Marianne B.M. van den Bree, Jonathan M. Green

**Affiliations:** 1 Division of Psychology and Mental Health, School of Health Sciences, University of Manchester, Manchester, UK; 2 Division of Psychological Medicine and Clinical Neurosciences, School of Medicine, Cardiff University, Cardiff, UK; 3 Manchester Centre for Genomic Medicine, Manchester University NHS Foundation Trust, Manchester, UK; 4 Neuroscience and Mental Health Innovation Institute, Cardiff University, Cardiff, UK; 5 Centre for Neuropsychiatric Genetics and Genomics, Cardiff University, Cardiff, UK

**Keywords:** 16p11.2 deletion, 16p11.2 duplication, Qualitative, Children, Families, Neurodevelopmental, Support, Experiences, Autism, Overshadowing

## Abstract

**Background:**

16p11.2 proximal deletion and duplication syndromes (Break points 4–5) (593KB, Chr16; 29.6-30.2mb - HG38) are observed to have highly varied phenotypes, with a known propensity for lifelong psychiatric problems. This study aimed to contribute to a research gap by qualitatively exploring the challenges families with 16p11.2 deletion and duplication face by answering three research questions: (1) What are parents’ perceptions of the ongoing support needs of families with children who have 16p11.2 living in the UK?; (2) What are their experiences in trying to access support?; (3) In these regards, do the experiences of parents of children with duplication converge or vary from those of parents of children with 16p11.2 deletion?

**Methods:**

33 parents with children (aged 7–17 years) with 16p11.2 deletion or duplication participated in structured interviews, including the Autism Diagnostic Interview– Revised (ADI-R). Their answers to the ADI-R question ‘what are your current concerns’ were transcribed and subsequently analysed using Braun and Clarke’s six step reflexive thematic analysis framework.

**Results:**

Three themes were identified: (1) Child is Behind Peers (subthemes: developmentally; academically; socially; emotionally); (2) Metabolism and Eating Patterns and; (3) Support (subthemes: insufficient support available; parent has to fight to access support; COVID-19 was a barrier to accessing support; 16p11.2 diagnosis can be a barrier to support, child is well-supported).

**Conclusions:**

Parents of children with either 16p11.2 deletion or duplication shared similar experiences. However, metabolism concerns were specific to parents of children with 16p11.2 deletion. The theme Child is Behind Peers echoed concerns raised in previous Neurodevelopmental Copy Number Variant research. However, there were some key subthemes relating to research question (2) which were specific to this study. This included parents’ descriptions of diagnostic overshadowing and the impact of a lack of eponymous name and scant awareness of 16p11.2.

## Background

Copy number variants (CNVs) are natural variations in the genome involving the deletion or duplication of a region of a chromosome, which, depending where in the genome they occur, may or may not have a pathogenic effect [1]. Neurodevelopmental CNVs (ND-CNVs) have been established to influence the likelihood of neurodevelopmental, mental health, and cognitive disabilities [2]. 16p11.2 proximal deletion and duplication syndromes (Break points 4–5) (593KB, Chr16; 29.6-30.2mb - HG38) are two such examples of ND-CNVs, and are prevalent in approximately 1/2000 and 1/1100 births respectively, in the general population [3]. ∼70% of 16p11.2 duplication carriers have inherited the CNV from a parent [4] whereas ∼ 29% of instances of 16p11.2 deletion are inherited [5]. 16p11.2 deletion and duplication syndromes are among the strongest known genetic risk factors for Autism Spectrum Disorder (ASD), with deletions and duplications of the BP4-5 region of chromosome 16 being prevalent in approximately 1 in 100 autistic individuals [6].

With regards to phenotype, 16p11.2 chromosomal rearrangements are associated with mirrored physical effects, with the deletion being associated with high body mass index, and the duplication a low body mass index [5, 7]. Similarly, whilst the deletion is associated with a large head circumference, the duplication is conversely associated with a small head circumference. Previous research has also investigated the psychiatric phenotype and has demonstrated that carriers of 16p11.2 deletion and duplication are at greater risk of having psychiatric conditions than controls. Up to 48% of deletion carriers and 63% of duplication carriers meet thresholds for one or more psychiatric condition, e.g. Attention Deficit Hyperactivity Disorder (ADHD), ASD, Anxiety Disorders, Oppositional Defiant Disorder, or Psychotic Symptoms [8, 9]. However, it is of note that 16p11.2 deletion and duplication are not fully penetrant, and as such, the resulting phenotype is highly variable between individuals [10]. Ongoing research strives to identify additional risk factors beyond the presence of the CNV, which might affect the observed phenotype, including common genetic variants and environmental risk factors [11].

The experiences of individuals and their families in the period of anticipation before receiving confirmation of a diagnosis of a ND-CNV has been previously explored. For example, 286 parents completed online surveys [12] and 57 parents completed interviews exploring their experiences during this period [13]. Their responses were analysed both qualitatively and quantitatively. This showed mixed views about the clarity of information offered from health professionals [12, 13] and conflicted feelings about the impact of receiving confirmation of a CNV, with many expecting both positive and negative implications for their child, e.g. more access to support but concerns about health problems [13]. Fitzgerald and colleagues [14] conducted a qualitative analysis of structured interviews with 30 parents of children who had recently received a rare genetic diagnosis in Ireland. They explored both parents’ understanding of the genetic result and the long-term impact they anticipated this would have on the wellbeing of the family. They identified key factors of wellbeing to be the clarity of language used to communicate the diagnosis, the availability of information and peer support, but the researchers did not report which specific ND-CNV diagnoses the participants had.

More specifically related to 16p11.2, Kleinendorst and colleagues [15] explored the experiences of families of children with 16p11.2 deletion in the Netherlands via focus groups with 23 caregivers from 16 families. Through thematic analysis they identified that families reported feelings of uncertainty during the diagnostic journey, barriers to accessing support services, a lack of understanding by others about what the diagnosis means, and their hopes for future support. However, there is a risk of over-representation of particular families’ views in this sample, with multiple caregivers from the same household participating in different focus groups. Furthermore, there is a need to explore whether the themes identified by families of 16p11.2 deletion carriers here echo those of families of 16p11.2 duplication carriers.

Furthermore, despite the known propensity for lifelong psychiatric conditions associated with the syndrome, there is less research focusing on the lived experiences of families after their child has been diagnosed with either 16p11.2 deletion or duplication. Whilst there is some work focusing on the lived experiences in the pre-diagnosis period, as far as we know, this is the first paper to qualitatively investigate the needs of both parents of children with 16p11.2 deletion or duplication, in the post-diagnostic period. This paper also expands the literature to explore the UK context.

## Methods

### Aim

This study aimed to explore, using qualitative methodology, the concerns experienced by families with children who have 16p11.2 deletion or duplication. More specifically we addressed three research questions: (1) What are parents’ perceptions of the ongoing support needs of families with children who have 16p11.2 living in the UK?; (2) What are their experiences in trying to access support?; (3) In these regards, do the experiences of parents of children with duplication converge or vary from that of parents of children with 16p11.2 deletion?

### Participants

37 parents or guardians, who had a child aged 7–17 years with a diagnosis of either 16p11.2 deletion (n = 17) or duplication (n = 20) syndrome took part, all were living in the UK. The data was drawn from two larger studies funded by the Medical Research Council (MRC) and National Institute of Health Research (NIHR) (see Funding section for full details) which were approved by the lead university’s research ethics committee - Cardiff School of Medicine, the National Health Service (NHS) East Midlands - Leicester Central Research Ethics Committee (19/EM/0287) and by all NHS ethics and research and development committees of participating UK Clinical Genetics Clinics. The two larger studies used a genotype-first approach, where recruitment was based on the presence of a genetic variant associated with increased risk of neurodevelopmental and mental health conditions, rather than a phenotype-first approach (based on the presence of neurodevelopmental and mental health conditions). The presence of the 16p11.2 deletion or duplication was confirmed from medical records and array genotyping in the laboratory of the Centre for Neuropsychiatric Genetics and Genomics at Cardiff University.

The inclusion criterion for this specific analysis followed opportunity sampling and required participants to have completed both of the above-named studies. We used the qualitative data from participants who had already completed a battery of interviews for us, as part of the wider study. We maximised the potential of the already collected data set, without fatiguing participants by asking further questions, as we were aware of the burden that more interviews would place onto them. The exclusion criterion was families who had insufficient English to preclude participation in structured interviews. The data were drawn from 44 participants who completed both of the above studies between April 2021 and December 2022. Seven of these parents were not included in this analysis: two were excluded by the research team due to family circumstances at the time of participation and the remaining five parents’ responses could not be transcribed sufficiently due to technological failures.

The 37 participants were recruited through a range of channels: from existing research databases (where consent to recontact had been provided), through UK Clinical Genetics Clinics, word of mouth, social media, and through charities and support groups for chromosomal disorders, including Unique [16]. Prior to participation, informed and written consent was obtained from all parents/carers. Young people over 16 years old also provided informed and written consent and children under the age of 16 informed and written assent. Parent/guardian respondents were predominantly female caregivers (33:4) and of the 37 children, 32 had at least one unaffected sibling. Further information about the demographics of the parent/guardian participants can be found in Table 1 and characteristics of their children in Table 2.

**Table 1:** Characteristics of parents/ guardians

Gender	Female	*n* = 33 (89%)
	Male	*n* = 4 (11%)
Age	Mean = 40.73 years	Standard Deviation = 7.01
Race	White	*n* = 35 (95%)
	Asian	*n* = 2 (5%)
Carrier of 16p11.2 del/dup	Carrier	*n* = 5 (14%)
	Non-carrier	*n* = 20 (54%)
	Untested	*n* = 12 (32%)
Highest Education Level	Secondary School (core educational provision up to 18 years old)	*n* = 16 (43%)
	Higher Education (additional studies resulting in national or degree level qualifications)	*n* = 21 (57%)
Current Employment	Full-time	*n* = 7 (19%)
	Part-time	*n* = 9 (24%)
	Unemployed/full-time carer	*n* = 21 (57%)

**Table 2:** Characteristics of children

Gender	Female	*n* = 20 (54%)
	Male	*n* = 17 (46%)
Age	Mean = 9.65 years	Standard Deviation = 2.50
Race (identified by parent )	White	*n* = 34 (92%)
	Asian	*n* = 1 (3%)
	More than one race	*n* = 2 (5%)
Country of residence	England	*n* = 30 (81%)
	Wales	*n* = 3 (8%)
	Scotland	*n* = 2 (5%)
	Northern Ireland	*n* = 1 (3%)
	Republic of Ireland	*n* = 1 (3%)
Inheritance of 16p11.2	Inherited	*n* = 9 (24.3%)
	De Novo	*n* = 12 (32.4%)
	Unknown	*n* = 16 (43.3%)
School Support	Mainstream + no additional support	*n* = 6 (16%)
	Mainstream + integrated support	*n* = 18 (49%)
	Separate class/specialist school	*n* = 11 (30%)
	Home-schooled	*n* = 2 (5%)
Parent reported Neurodevelopmental/ Psychiatric Diagnoses	Learning Disability	*n* = 22 (59%)
	Developmental Delay	*n* = 22 (59%)
	ASD	*n* = 11 (30%)
	ADHD	*n* = 6 (16%)
	Anxiety	*n* = 3 (8%)
	Depression	*n* = 0 (0%)
	Psychosis	*n* = 0 (0%)
	Bipolar Disorder	*n* = 0 (0%)
	OCD	*n* = 0 (0%)

### Procedure

As mentioned above, the parents/guardians were recruited and consented into the wider studies from April 2021 - December 2022. As part of these studies, they participated in structured interviews, including the Autism Diagnostic Interview- Revised (ADI-R) [17] conducted within the home environment or via zoom calls.

Parents’ answers to the question *‘do you have any current concerns about CHILD’s behaviour or development’* were transcribed verbatim by CB and CG. Researchers used up to 3 extra prompts to gather additional information about the experiences shared by parents. Examples of prompts included: what was that like? Or ‘when was that?’, as specified within the ADI protocol.

### Analysis

The analysis was conducted by CB and CG using Braun and Clarke’s 6-step reflexive thematic analysis framework [18, 19]. This was chosen because the aim of the analysis was to be data-driven when depicting the experiences of families with children diagnosed with 16p11.2 deletion or duplication in the UK. A deductive, or top-down, approach was used to code the excerpts to see if the experiences of UK parents reflected the findings of previous research conducted outside the UK. The research team acknowledges that our positionality is influenced by working closely with the families involved and wanting to help them to share their experiences. We also acknowledge that we do not have lived experience of 16p11.2 deletion or duplication. We identified the themes at a semantic level, within a realist/essentialist framework. Therefore, we accepted what participants said to be the reality, rather than exploring how their reports were driven by the socio-cultural context. We maintained our focus on identified current concerns and not the concerns at a systemic level i.e., differences in provision across the UK local authorities or health trusts. This data-driven analysis enabled a broad examination from different perspectives that was not shaped by any pre-determined categories or question prompts. We acknowledge that there may be elements of social desirability bias towards the researchers. However, these families were working with the research team for multiple sessions, building rapport, and providing them with an outlet to tell the truth about their experiences.

CB and CG separately familiarised themselves with the excerpts and subsequently collaboratively assigned codes. They then listed all codes and built these into initial themes, after which the distinguishability of the initial themes was assessed and re-developed accordingly. Any discrepancies were discussed with KL and resolved through consensus to form the final three themes and sub-themes outlined in Table 3. Building the themes collaboratively (CG and CB) and having them moderated by KL ensured credibility and confirmability of the results. All analysts hold at least a relevant undergraduate degree (CB, CG) or PhD (KL) and were working directly with families with 16p11.2 deletion or duplication (CB & CG) or within intervention research for neurodiverse families (CB, CG & KL).

**Table 3:** Definitions of themes and sub-themes

Theme	Definition of Theme	Sub-themes	Definition of Sub-themes
Child is behind peers	Parent expresses concerns that there is a gap between child and their peers	Developmentally	Parent is concerned about child’s developmental delay
		Academically	Parent is concerned child is academically behind peers
		Socially	Parent is concerned about child’s communication and navigation of social interactions with peers
		Emotionally	Parent feels child has more difficulty with emotional understanding than peers
Metabolism and eating patterns	Parent expresses concerns about child’s eating, weight gain or metabolism	No sub-themes	
Support	Parent describes the level of support child has and journey to receiving this	Insufficient support available	Parent describes areas where child does not receive enough support
		Parent has to fight to access support	Parent feels accessing support requires a battle
		COVID-19 was a barrier to accessing support	Parent feels COVID was the cause of difficulties in accessing support
		A 16p11.2 diagnosis can be a barrier to support	Parent feels 16p11.2 diagnosis prevents a deeper exploration of child’s needs
		Child is well-supported	Parent describes areas where child receives a good amount of support

## Results

CB and CG looked for patterns in the data to differentiate the experiences of parents of children with 16p11.2 deletion from those of parents of children with 16p11.2 duplication but found that parents of children with both CNVs converged on most themes, with concerns about metabolism and weight being the only exception. To protect anonymity all names and pronouns were excluded from the quotations below.

### Child is behind peers

A key theme we identified, shared by both parents of children with 16p11.2 deletion and duplication, was parents’ worries about the gap between their child and their peers. We split this into four sub-themes:

#### Developmentally

Many parents expressed concerns about the development of their child in comparison to their peers:

CHILD’s brain is kind of like 4–5 years behind in my view.

(52-year-old female)

This parent’s response suggests that the delay they observe between their child and their child’s peers is significant, with their child behaving like, and having the abilities of, a much younger child.

Other parents made similar observations, for example:

CHILD will trust anyone and they are extremely vulnerable and we can’t let them out of our sight.

(41-year-old female)

This parent is identifying that their child’s awareness of the world is significantly behind their peers and that they feel this inhibits opportunities for independence.

#### Academically

For other parents, the focus of their concerns was more specifically linked to their child’s academic progress:

at the moment definitely CHILD’s academic learning. So that is one of them, CHILD really is struggling learning-wise.

(40-year-old female)

In line with this parent, many parents felt that their child struggled to focus on academic goals and that accessing learning was more challenging for their child than their child’s peers.

#### Socially

Another element of concern was gaps in communication and navigation of social situations compared to peers:

CHILD’s speech really, not being able to pronounce their words, because fundamentally, if they could talk more they could communicate better and I think that would help.

(39-year-old female)

This parent expressed their distress around the challenges their child experiences in being unable to clearly communicate to others. Other parents also mentioned specific concerns about the impact their child’s gap from peers has on friendships:

There is a huge friendship problem and CHILD really struggles to make friends.

(40-year-old female)

This parent explained that building friendships with peers is more of a challenge for their child than for their child’s peers.

#### Emotionally

Finally, the difficulties in navigating emotions in comparison to peers was a multifaceted topic of concern. Firstly:

CHILD is finding it hard to convey their emotions, how CHILD is feeling.

(40-year-old female)

For some parents the essence of their child’s communication difficulties was that their child was unable to find the words to explain their emotions and internal experiences. Other parents felt their child also struggled to understand the reasons behind their feelings and behaviour:

When CHILD is low they don’t know why they’re low and CHILD would in their head think of the first thing that then makes them sad and says I’m sad about this and actually looking at the pattern that’s not why CHILD’s sad.

(41-year-old female)

This parent explains that they have observed that their child struggles with interoception, which is understood as difficulty in interpreting the internal state of their body. Their child has learned to use a term to describe these feelings to be able to answer others but in reality they do not really understand the causes of their feelings.

### Metabolism and eating patterns

We found this theme to be of concern solely to parents of children with 16p11.2 deletion, who reported worries about their child’s eating behaviours and weight:

CHILD was really struggling with eating and putting on loads of weight, we think it’s 16p11.2.

(49-year-old male)

This parent expresses worries about the volume of food their child consumes and feels this pattern of eating behaviours is related to their 16p11.2 deletion diagnosis. Other parents shared similar concerns, but were most worried about metabolism:

Our biggest concern through the whole lot would be CHILD’s metabolism.

(48-year-old male)

This parent felt that their child’s weight gain was not necessarily a reflection of their eating behaviours and thought that rather it was caused by a change in the way their child’s body processed the food that they ate.

### Support

Finally, a recurring and important theme that was identified to be common across parents of children with both 16p11.2 deletion and duplication was sharing their experiences of their child’s level of support and the journey to receiving this. We divided this into five subthemes: insufficient support available; parent has to fight to access support; COVID-19 was a barrier to accessing support; a 16p11.2 diagnosis can be a barrier to support; child is well-supported.

#### Insufficient support available

Many parents described areas where they worry their child does not receive enough support. For some parents this focused on support from health professionals:

that’s how our health board they say that you only get speech and language for two years and then magically, you’re fixed, but what where you’ve got a congenital condition that’s gonna be, you know, for life yeah, you know two years doesn’t do it.

(50-year-old male)

This parent indicated that their local services do not understand the impact of a life-long condition such as 16p11.2 deletion or duplication syndromes and that, as a consequence, the available support feels time-stamped. The concerns of other parents were focused on support for their child’s mental health:

*CHILD’s been there and discharged and then back there and discharged twice and it’s like one step forward and five steps back with CAMHS [Child and Adolescent Mental Health Service]…we never get any follow ups from anywhere*.

(34-year-old female)

This parent expressed strong feelings of frustration and concerns they were often left to cope with their child’s difficulties alone. This sentiment was often shared by the other parents in our sample.

#### Parent has to fight to access support

Many parents felt they had to undertake a lot of responsibility in fighting to access support for their child:

We’re in the middle of a battle with the local authorities to get a EHCP [Education, Health and Care Plan] in place. I do feel that CHILD needs a lot more support than CHILD is getting at the moment in school.

(40-year-old female)

This parent highlights the seriousness of their concerns using the word ‘battle’ to emphasise the challenges they face in meeting the educational needs of their child. Similarly:

It was so so difficult for years to try and get professional people to understand that CHILD’s not, you know, falling into that category but it doesn’t mean CHILD doesn’t need support.

(39-year-Old female)

This parent explained that part of their fight to access support is a consequence of their child not being disruptive which meant that professionals overlook their child’s needs.

Other parents expressed similar concerns with accessing support for their child’s physical health:

*It’s me that’s always like pushing, you know, for things for CHILD, you know, what if i wasn’t as clued up as I am, you know, I’m constantly going on at everyone, you know, check CHILD’s blood, check CHILD’s, you know, CHILD’s cholesterol, check is it check CHILD’s weight, check it, you know, check everything, you know, it’s me just badgering all the time, you know, but nobody ever wants, you know, nobody’s ever looking, it’s me shouting for the dieticians, you know, to come, you know, it’s just me constantly harassing people, you know*.

(47-year-old female)

This parent describes the role parents have to play, using the strong word choice of “harassing”, and feeling this level of persistence is necessary in order to get people to listen to their concerns.

#### COVID-19 was a barrier to accessing support

Given the temporal context of this project, the COVID-19 pandemic appeared as a recurring obstacle for parents in accessing support for their children, with some parents acknowledging the delays the pandemic caused:

everything ground to a halt because of covid so we are now starting again to get in contact with the agencies that we should have been in contact with for the past two years but weren’t able to with covid because everything just shut down so it is like back to square one again.

(48-year-old female)

This parent identifies the pandemic and subsequent lockdowns as the root of the delays that they had experienced recently in accessing support from school and health professionals. Other parents shared similar experiences:

Yeah CHILD was under the CAMHS team for anxiety but because of COVID and everything and the courses that they offered were kind of relevant when the course was prescribed to them but then because of COVID, CHILD is now too old.

(40-year-old female)

For this parent, the delays caused by the COVID-19 pandemic meant that their child was no longer eligible for the support that was available in their local area.

#### A 16p11.2 diagnosis can be a barrier to support

Another barrier to support that was commonly identified as a recurring concern of our parents, is the lack of understanding about 16p11.2 as a condition. Parents felt professionals often overlooked comorbidities once the diagnosis of 16p11.2 deletion or duplication was confirmed. For example:

CAMHS have said CHILD’s not for them, they don’t want to assess CHILD for autism because CHILD’s only got a few traits they say CHILD hasn’t got enough traits so it’s kind of because CHILD’s got this genetic condition they’re not willing to look into anything else that could be alongside it.

(32-year-old female)

This parent felt that because their child has a diagnosis of 16p11.2, professionals are reluctant to explore the autistic traits the parent has observed further. Similarly:

obviously CHILD’s got this diagnosis of the chromosome disorder but we feel there’s something else out there as well and we can’t seem to get anyone to listen to us to kind of investigate any other kind of things that CHILD might have.

(41-year-old female)

This parent echoed the concerns above and feels that their child would benefit from further assessments but found that it is difficult for this need to be heard by professionals.

Some of the participating parents felt this barrier was linked to a lack of awareness about what 16p11.2 deletion and duplication syndromes are:

nobody knows anything about it really from a medical point of view well, CHILD has 16p11.2 I really do think CHILD’s got autism but they’re not bothered, it might be 16p11.2, it might be autism, we can’t really diagnose it…who do I talk to? You know if CHILD’s got other conditions I can go and see someone about this, CHILD’s got all these conditions but with 16p11.2 the best I can speak to is a geneticist but they’re not really the 16p11.2 expert, they’re just a geneticist. So I think, I do think that’s missing from 16p11.2.

(49-year-old male)

This parent acknowledged that a 16p11.2 diagnosis stopped them from accessing further assessments. They wished there was a wider network of specialists who could give answers that are specific to the needs of families of children with 16p11.2 deletion or duplication.

#### Child is well supported

Finally, despite the barriers and challenges in accessing support, some parents did report that they felt their child’s needs were being met, e.g. within the family unit:

*“we’re so acutely aware of what CHILD’S challenges are because that’s what I’ve always sort of done as a job so we’ve adapted our environment and you know, we’ve never really struggled with any of that sort of stuff”*.

(40-year-old female)

This parent explains that personal experience has supported them to adapt the family routines to meet their child’s needs. Similarly:

School have been amazing though they have already incorporated aspects of the framework for learning for autism in CHILD’s day to day life at school so that has been one area where we have actually had progress.

(48-year-old female)

This parent was pleased by the support that was accessible for their child in school and felt that this had been a great help.

## Discussion

We identified three themes from the responses shared, which included: concerns that children were behind their peers; concerns about metabolism and eating patterns; and concerns or praise for the level of support received from a variety of professionals.

**(1): What are parents’ perceptions of the ongoing support needs of families with children who have 16p11.2 living in the UK?**

Some of the sub-themes we identified echoed the experiences of carriers of a wider range of ND-CNVs. For example, the theme “child is behind peers” is a common worry shared by parents of children with a variety of ND-CNVs, as the differences that they experience can cause challenges across their everyday lives [20, 21]. Previous literature has reported that a third of children with 16p11.2 deletion or duplication have an Intellectual Disability [8]. Our sample showed higher rates with 59% of children parentally-reported to be diagnosed with Global Developmental Delay, Learning Disability or both (Table 2). Therefore, parents’ worries about their child being delayed in comparison to peers were not unexpected. Previous research supports the views of parents in our sample that communication difficulties need to be supported to prevent this becoming a barrier to socialising with peers, [15] which could suggest that this would be a key focus for future early-intervention.

**(2): What are their experiences in trying to access support?**

Overall, both within and across families, support was described to be varied and inconsistent. It was often the case that parents in our sample would feel support was strong in one area and lacking in others, which was a theme also reported by parents in the Netherlands [15].

Furthermore, parents in our sample reported that the complex and sometimes life-long impacts are not sufficiently addressed by offers of routine support and expressed a wish for increased support in the post-diagnostic period. This is consistent with the findings of studies with parents across a wider range of ND-CNVs [14].

Due to the temporal setting of the study, parents further identified the COVID-19 pandemic and lockdowns to be a barrier to accessing any support that should have been available due to the disruption this caused to support services. Indeed, in the UK it was estimated that 1 in 35 individuals were waiting to receive specialist treatment from the NHS in 2022 [22]. Therefore, this is an expected challenge that will continue for the foreseeable future across all sectors of care.

Whilst parents of 16p11.2 deletion/ duplication carriers shared common concerns with other ND-CNV parents with regards to research question (1), there were some key sub themes relating to research question (2) which emerged from this specific study. For example, we identified that parents felt that knowledge of 16p11.2 deletion/ duplication in the public eye is extremely limited and in some professionals may appear scant, and as such, that parents felt the need to fight for support for their children. Parents chose to use words such as “harassing” and “battle”, when describing accessing support, emphasising how challenging they feel the process of gaining support to be, a finding that was also reported by Kleinendorst and colleagues [15].

To mitigate this need to fight to break down barriers, parents of carriers of other ND-CNVs identified peer networks, or advice found online, to be a key source of support [14, 23, 24]. This was not mentioned by parents in our sample and may reflect a gap in support for these parents, with no charity specifically focusing on 16p11.2 deletion or duplication syndromes in the UK. Previous literature has identified that fighting for support as a parent of a child with a rare genetic condition can have a negative emotional impact [25, 26]. Therefore, there is also a clear need to improve and standardise access to support, to identify lead professionals and to build parent support networks.

Parents felt that a 16p11.2 deletion or duplication diagnosis and their varied phenotypes can themselves be a barrier to accessing support as this reduces clinicians’ willingness to explore possible co-occurring psychiatric conditions in depth. It has been described, in the context of Learning Disability, that co-occurring psychiatric or neurodevelopmental conditions can go undetected due to over-attribution of symptoms to a primary diagnosis [27]. This over-attribution has been labelled ‘Diagnostic Overshadowing’ [28] and from the description given by parents we suggest that it would be important to consider the role this plays in disentangling the comorbidities observed in 16p11.2 deletion and duplication. Kleinendorst and colleagues [15] built on this to suggest that more specifically, the unfamiliarity of 16p11.2 deletion syndrome and the lack of an eponymous or memorable name was a barrier to accessing support. Therefore, it is imperative that work continues to raise awareness of the profile of 16p11.2 deletion and duplication syndromes and their phenotypic presentations in order to avoid this diagnostic overshadowing, which is described by parents.

**(3): In these regards, do the experiences of parents of children with duplication converge or vary from that of parents of children with 16p11.2 deletion?**

Parents of children with 16p11.2 deletion differed from parents of children with 16p11.2 duplication with their worries about their child’s metabolism and eating patterns. Their concerns were supported by previous research that identified a link between 16p11.2 deletion and higher Body Mass Index ratings (BMI) caused by a change in metabolic rate [29] or alterations in satiety signals [30]. Conversely, 16p11.2 duplication syndrome is linked to low BMI [5] so the fact that weight gain concerns were only expressed by parents of children with 16p11.2 deletion is an expected finding.

Nevertheless, parents of children with 16p11.2 deletion and duplication collectively identified the same gaps between their children and peers and the same inconsistencies regarding availability of support. This suggests that overall they shared similar experiences and similar barriers.

### Limitations

This study was conducted as part of a wider study. As a result, the participants were not recruited with this study’s specific research questions in mind. The questions asked were not designed to elicit detailed descriptions about individual experiences, therefore we acknowledge that the answers given to our structured interview questions might be less comprehensive than answers to bespoke interview questions. Despite a nationwide recruitment net, involving multiple modalities, our findings might be subject to ascertainment bias as inclusion criteria for the wider study included that participating carriers had received a clinical diagnosis of 16p11.2 deletion or duplication. Given the possibility that many children with 16p11.2 CNVs remain undiagnosed, it is possible that the findings will be representative of families who had experienced a more severe phenotype that had prompted clinical investigation. Another limitation is the temporal setting of this study with the backdrop of the Covid-19 pandemic which may have limited families’ willingness to participate in a research study requiring lengthy interviews, linked to zoom fatigue or elevated Covid-19 -related stress levels that lowered general capacity to take part. Furthermore, we acknowledge limits in transferability, with some themes specific to this temporal setting, as well as other concerns raised by parents being specific to the UK setting (including CAMHS, NHS). Additionally, the sample mostly captured the views of female parents and lacked racial diversity so cannot claim to be fully representative of all parenting experiences. Finally, although our sample did include families from all four nations of the UK, wider representation internationally would be beneficial to fully evaluate the influences of systemic differences linked to the needs of families with children who have 16p11.2 deletion or duplication.

### Recommendations for future research

Based on our findings, future research should look to expand on the lived experiences of families with 16p11.2 deletion or duplication, using specific questions to elicit more detailed answers that can be used to understand both individual experiences and systemic factors. Systemic research should also encompass greater representation across the four countries of the UK to identify themes that would support best practice going forwards. A further area of future research could be to explore whether parental representations differ between families of inherited 16p11.2 deletion or duplication, compared to de novo. It would also be important to improve racial and parental diversity to increase representation and to explore how this impacts differences in experiences.

### Clinical implications

The findings of this paper support the importance of understanding families’ perspectives, given that ND-CNVs are an increasingly recognised type of genetic mutation that are frequently seen within clinical settings. Our findings reinforce the ongoing need to provide individually tailored education and communication support to children with ND-CNVs. There is also a clear need to improve and standardise access to support, to identify lead professionals and to build parent support networks, which has previously been identified to be a protective factor [14, 23]. Finally, some specificity in care, with regards to dietary guidance, may be valuable for the children, and parents of children, with 16p11.2 deletion.

## Conclusions

Parents in our sample felt that their difficulties with accessing support extended beyond the common experiences of parents of children with other ND-CNVs and suggested that 16p11.2 deletion and duplication comes with an additional challenge. We think it is important to consider the role that a lack of eponymous or memorable name might play in creating additional barriers for parents and professionals to navigate [23] and the influence this may have on levels of understanding and consequent diagnostic overshadowing. Additionally, we found that overall, aside from syndrome specific symptoms relating to weight and metabolism, both parents of children with 16p11.2 deletion and duplication shared similar experiences and concerns, which helps to suggest that the gap in the literature of 16p11.2 duplication voices can be supported by existing evidence from 16p11.2 deletion voices. However, hearing the experiences shared by parents in our sample has also reinforced that the varied phenotype of 16p11.2 deletion and duplication means personalised care and peer support should always be a priority to adequately meet the needs of families.

## Data Availability

Data are held at Cardiff University. The data are available upon request. Please contact Prof. van den Bree (vandenbreemb@cardiff.ac.uk) for any data requests.

## Declarations

### Authors’ information

JG is a NIHR Senior Investigator (NF-SI-0617-10168) and supported by the NIHR Manchester Biomedical Research Centre (NIHR203308). The views expressed are those of the authors and not necessarily those of the NIHR or the Department of Health and Social Care.

## Abbreviations

CNVs: Copy Number Variants
ND CNVs: Neurodevelopmental Copy Number Variants
ASD: Autism Spectrum Disorder
ADHD: Attention Deficit Hyperactive Disorder
NHS: UK National Health Service
CAPA: Child Adolescent Psychiatric Assessment
SIPs: Structured Interview for Prodromal Syndromes
ADI-R: Autism Diagnostic Interview Revised
CAMHS: UK Child Adolescent Mental Health Service
EHCP: Education, Health and Care Plan, England specific
BMI: Body Mass Index
